# Association of the genetic ancestry with resistant hypertension in the ReHOT (Resistant Hypertension Optimal Treatment) randomized study

**DOI:** 10.1038/s41598-020-58540-3

**Published:** 2020-01-30

**Authors:** Carolina Tosin Bueno, Alexandre Costa Pereira, Hadassa Campos Santos, Luz Marina Gómez Gómez, Andrea Roseli Vançan Russo Horimoto, Eduardo Moacyr Krieger, Jose Eduardo Krieger, Paulo Caleb Junior Lima Santos

**Affiliations:** 1 Laboratory of Genetics and Molecular Cardiology, Heart Institute, University of Sao Paulo Medical School, São Paulo, Brazil; 2 Department of Pharmacology – Escola Paulista de Medicina, Universidade Federal de Sao Paulo EPM-Unifesp, São Paulo, Brazil

**Keywords:** Genetic testing, Cardiovascular genetics

## Abstract

Resistant hypertension (RH) is defined as uncontrolled blood pressure despite treatment with three or more antihypertensive medications, including, if tolerated, a diuretic in adequate doses. It has been widely known that race is associated with blood pressure control. However, intense debate persists as to whether this is solely explained by unadjusted socioeconomical variables or genetic variation. In this scenario, the main aim was to evaluate the association between genetic ancestry and resistant hypertension in a large sample from a multicenter trial of stage II hypertension, the ReHOT study. Samples from 1,358 patients were analyzed, of which 167 were defined as resistant hypertensive. Genetic ancestry was defined using a panel of 192 polymorphic markers. The genetic ancestry was similar in resistant (52.0% European, 36.7% African and 11.3% Amerindian) and nonresistant hypertensive patients (54.0% European, 34.4% African and 11.6% Amerindian) (p > 0.05). However, we observed a statistically suggestive association of African ancestry with resistant hypertension in brown patient group. In conclusion, increased African genetic ancestry was not associated with RH in Brazilian patients from a prospective randomized hypertension clinical trial.

## Introduction

Hypertension is one of the predominant driver of cardiovascular diseases^1^. Many studies have correlated the predisposition to hypertension with African ancestry^2–5^. In particular, apart from the association with Black race (which has been extensively described), much discussion persists on the reasons for this association. For one point, socioeconomically inequalities are pervasive in most Western populations and known to be very difficult to adjust for in epidemiological studies^6^. Nonetheless, several lines of evidence also point to a higher frequency of potentially deleterious alleles in individuals with high African ancestry, leaving the question of into what degree the association between hypertension and race is biologically defined^7^. This may have wide impact on the future management of hypertension from a medical standpoint, as well as in the development of health policies targeted as reducing the impact of chronic disease in the general population.

Resistant hypertension (RH) is defined by uncontrolled blood pressure despite treatment with three or more antihypertensive medications, including, if tolerated, a diuretic in adequate doses^8,9^. Patients with RH have even higher risk of cardiovascular complications than hypertensive patients^10^. It is estimated that 10–20% of people with hypertension are resistant, underscoring the importance of understanding the determinants of this condition^11–13^.

In Brazil, self-declared race/color is based on a complex subjective phenotypic and cultural assessment because the Brazilian population is highly admixed with the main ancestral contributing populations being European, African and Amerindian^14,15^. Of particular importance to the present work most Brazilians are defined as a mosaic of these three main ancestries and a much higher degree of discordance between self-referred race and genetic ancestry is seem as compared to the US^16–18^.

In this scenario, we have an interesting hypertensive population to evaluate, with systolic blood pressure >160 mmHg and <220 mmHg, and/or diastolic >100 mmHg, in the seated position^19^. These measurements were taken until two consecutive measurements differing less than 4 mmHg between them, with a very controlled methodology and prospectively, being a robust way to identify RH patients. Thus, with a significant number of diagnosed RH patients, we sought to evaluate a possible association of genetic ancestry with RH in a large multicenter prospective randomized clinical trial from the ReHOT study^20^.

## Material and Methods

### Study population

The patients come from a prospective randomized clinical trial (Resistant Hypertension Optimal Treatment - ReHOT) that was conducted between 2012 and 2014. It included hypertensive patients from 26 sites located in different states in Brazil^20^. The Ethics Committee for Human Medical Research of the Clinical Hospital of the School of Medicine, University of São Paulo approved the study protocol (protocol number 0758/09) and all individuals signed an informed consent form in accordance with the Declaration of Helsinki and good clinical practice guidelines. This study was registered at clinicaltrials.gov under the number NTC1643434.

#### Genotyping data

Individuals from the ReHOT project were genotyped using genomic DNA in the Open Array platform with QuantStudioTM 12 K Flex^TM^. A commercial genotyping custom chip from Applied Biosystems^TM^, containing 192 single nucleotide polymorphisms (SNPs which can be seen in Supplementary Table 1), was used to perform genotyping through real-time PCR. In this method, each chip had enough arrays to obtain the 192 genotypes of 15 individuals at the same time and a further negative control reaction to evaluate the quality of genotyping. Estimates of individual admixture using this 192 AIM panels were highly correlated with estimates using ~370 000 genome-wide SNPs: 91%, 92%, and 74% of, respectively, African, European, and Native American ancestry components^21^.

#### Genetic ancestry

Ancestry informative markers (AIM) are important tools to differentiate populations and estimate proportions of ancestry. These analyze are based on genetic ancestry (GA) whose allelic polymorphisms frequencies vary markedly in different racial and ethnic groups and they can be used to control wrong results from stratifications and analyses by race^22^. The implementation of the panels of AIM, in platforms high-throughput genotyping, would facilitate simultaneous mapping of characteristics of complex diseases^23^.

The analysis of GA was performed using the Admixture program^24^, which is a tool for estimating the maximum likelihood of individual ancestors of data sets of *multilocus* SNP genotypes. The ancestry proportions were estimated for our samples using the panel of AIMs and samples from HapMap, HGDP and 1000 Genomes projects as reference for European, African and Native American populations^21^.

The reference populations used for African were ASW, LWK and YRI from HapMap3 (Americans of African Ancestry in SW USA; Luhya in Webuye, Kenya; Yoruba in Ibadan, Nigeria); Bantu and Mandenka from HGDP (Sub-Saharan Africa). The populations used for European were CEU and TSI from HapMap3 (Utah Residents with Northern and Western European ancestry; Tuscany in Italy); GBR and IBS from 1000 Genomes (British in England and Scotland; Iberian population in Spain) and Orcadian, Russian from HGDP (Northern Europe). In the end, the populations used for Amerindian were MEX from 1000 Genomes (Mexican Ancestry from Los Angeles USA); Pima, Maya, Karytiana and Surui from HGDP (Mexico and Brazil). After choosing the populations, the GA of each patient in the Admixture program was calculated^24^.

#### Statistical analysis

Manipulation of the genotypic database was done by the PLINK program^25^. For the statistical analysis, we used the R program (version 3.0.0). Chi-square or Fisher’s exact tests were used to compare categorical variables. Resistant hypertension was used as a binary variable. T-test, Mann-Whitney, Anova and Kruskal-Wallis were used to determine differences between the means of numerical variables.

## Results

Samples from 1,358 patients were analyzed for the proportions of EUR, AFR and AMR ancestry. From these, 165 were resistant hypertensive patients. Table 1 shows general and clinical characteristics of patients according to self-declared race/color groups. The average age was 54.7 years for whites and 52.9 years for non-whites. The mean blood pressure was 172.5/102.6 mmHg and 42% of the patients were males and 58% females. As expected there was a highly significant association between genetic ancestry (GA) and self-declared race/color (Supplementary Table 2). Table 2 shows that the proportion of ancestry was not associated with resistant or nonresistant hypertensive patients. The GA proportions were similar in both groups with 52.0% European, 36.7% African and 11.3% Amerindian for resistant and 54.0% European, 34.4% African and 11.6% Amerindian (p > 0.05) for nonresistant patients.

**Table 1:** General and clinical characteristics among self-declared race/color groups of patients.

Variables	White N = 575	Non-white N = 783	p value	CI
**Age (years)**	54.7	52.9	0.001	(−2.908; −0.738)
**Body mass index (kg/m**^**2**^**)**	30.0	31.1	0.115	(−0.134; 1.224)
**Gender (%)**				
Men	43.1	40.6	4.02e^−05^*	(0.477; 0.774)
Women	56.9	59.4	2.00e^−08^*	(0.402; 0.607)
**Education level (%)**				
Illiterate	2.6	4.5	0.0001*	(0.069; 0.464)
Elementary school	77.0	85.4	2.00e^08^*	(0.368; 0.521)
Higher education	20.4	10.1	0.0001*	(1.437; 3.363)
**Alcohol use (%)**				
Yes	33.4	37.3	2.02e^05^*	(0.333; 0.568)
No	66.6	62.7	3.17e^−07^*	(0.501; 0.738)
**Smoker (%)**				
Non-smoker	60.9	60.4	2.16e^−08^*	(0.448; 0.669)
Former smoker	29.5	30.4	2.61e^06^*	(0.382; 0.680)
Smoker	9.6	9.2	0.044*	(0.343; 0.987)
**Congestive heart failure (%)**				
Yes	0.3	0.8	0.132**	(0.004; 1.568)
No	99.7	99.2	2.00e^08^	(0.465; 0.635)
**Stroke (%)**				
Yes	3	4.2	0.0025*	(0.104; 0.651)
No	97	95.8	2.00e^−08^*	(0.472; 0.648)
**Acute myocardial infarction (%)**				
Yes	0.9	1.1	0.257*	(0.044; 1.860)
No	99.1	98.9	2.00e^−08^*	(0.464; 0.634)
**Diabetes mellitus (%)**				
Yes	18.1	16.1	0.050^*^	(0.463; 1.000)
No	81.9	83.9	2.00e^−08^*	(0.433; 0.609)
**Dyslipidemia (%)**				
Yes	32.2	26.2	0.174	(0.608; 1.089)
No	67.8	73.8	2.00e^−08^	(0.378; 0.548)
**Obstructive heart failure (%)**				
Yes	1	0.3	0.132**	(0.638; 2.780)
No	99	99.7	2.00e^−08^*	(0.454; 0.620)
**Systolic BP at study entry (mmHg)**	171.3	173.6	0.011	(0.492; 3.877)
**Diastolic BP at study entry (mmHg)**	102.1	103.0	0.116	(−0.245; 2.230)

*Teste chi-square test.

**Fisher’s exact test.

**Table 2:** Association of the genetic ancestry with resistant or nonresistant hypertension patients.

Genetic ancestry	Nonresistant N = 1,193	Resistant N = 165	p value
European	0.540	0.520	0.304
African	0.344	0.367	0.257
Amerindian	0.116	0.113	0.680

A stratified analysis was performed on two groups (white and non-white) (Table 3). The group of non-whites (black and brown patients) had 684 nonresistant and 99 resistant patients, whereas the group of whites had 509 nonresistant and 66 resistant patients, but no significant difference was found between these groups.

**Table 3:** Association of the genetic ancestry with resistant or nonresistant hypertension among white and non-white patients.

Genetic ancestry	White (N = 575)		
	Nonresistant N = 509	Resistant N = 66	p value
European	0.711	0.709	0.936
African	0.174	0.174	0.918
Amerindian	0.115	0.117	0.710
	**Non-White (N = 783)**		
**Genetic ancestry**	**Nonresistant N = 684**	**Resistant N = 99**	**p value**
European	0.413	0.391	0.320
African	0.471	0.501	0.244
Amerindian	0.116	0.108	0.427

In an analysis stratified by white, brown and black, no significant association of the GA with RH was observed (Supplementary Table 3). However, we observed a statistically suggestive association of African ancestry with resistant hypertension in brown patient group in a model shown in Supplementary Table 4. Thus, we evaluated a possible association of general variables on African ancestry (dividing into 2 groups among African ancestry median), in this brown group. We did not find association of general variables with African ancestry in this analysis (Supplementary Table 5).

## Discussion

The ReHOT study identified 11.7% of hypertensive patients that fulfilled the RH criteria. In the study, there was no association between self-declared race/color with RH^26^. In the present study, our hypothesis of a possible association of genetic ancestry with RH was not confirmed.

Some studies showed an association of black race/color with higher prevalence of RH patients. One of them showed a prevalence of RH of 7.3% in European Americans, while there was a greater proportion of RH patients in African Americans (10.6%)^27^. Another study, developed in an ethnically and socioeconomically diverse population from California, identified a RH cohort of 12.8% from hypertensive patients. It found that they were more likely to be black^8^. Some variables, such as advanced age, increased body mass index, male gender, genetic variants and others chronic diseases were also associated with RH or uncontrolled hypertension^28–31^.

On the other hand, some studies, including the present study, did not find an association of self-reported race/color with RH. A study with 9,361 hypertensive patients from the US, did not find differences in RH proportions according to race^32^. In addition, a meta-analysis, which analyzed the global prevalence of RH from data of 3.2 million patients, did not observe differences^33^. Some studies have observed genetic variants associated with risk for resistant hypertension^2,34,35^. However, to the best of our knowledge, studies of genetic ancestry and RH are not identified in the literature. Our hypothesis was that an increased proportion of African ancestry might be a risk factor for RH, in the overall group or even in subgroups among self-declared race/color, but this was not confirmed. However, in brown patient group, we identified a statistically suggestive association of African ancestry with resistant hypertension. This statistically suggestive association suggests that the criteria applied to segregate the race/color groups, according Brazilian Institute of Geography and Statistics, are possibly distorting the real distribution and, at least in part, leading a limitation for dividing into homogeneous groups. Thus, further investigations and new hypothesis should be tested to elucidate possible associations.

The prevalence and incidence of RH will grow considerably because there is a large number of overweight/obese individuals^36^. In addition, the cutoff blood pressure criteria for RH was reduced from 140/90 mmHg to < 130/80 mmHg^9^. Thus, prevent damages by RH and identify better treatments is important, because the number of RH patients can increase with this new cutoff^37–40^. A possible limitation of our study is the low statistical power to differentiate mean values of African ancestry among patient groups.

## Conclusion

In the present study, genetic ancestry was not associated with resistant hypertension in Brazilian patients from a prospective randomized clinical trial.

## Supplementary information

Supplementary infomation.
