# Supplementary material for: Association of the genetic ancestry with resistant hypertension in the ReHOT (Resistant Hypertension Optimal Treatment) randomized study
Source: Sci Rep. 2020 Jan 30;10:1476. doi: 10.1038/s41598-020-58540-3 (PMC6992613; doi:10.1038/s41598-020-58540-3)
Supplement: Supplementary file 1 — Supplementary infomation. [file 41598_2020_58540_MOESM1_ESM.docx]

**Association of the genetic ancestry with resistant hypertension in the ReHOT (Resistant Hypertension Optimal Treatment) randomized study**

### Carolina Tosin Bueno^1^, Alexandre Costa Pereira^1^, Hadassa Campos Santos^1^, Luz Marina Gómez Gómez^1^, Andrea Roseli Vançan Russo Horimoto^1^, Eduardo Moacyr Krieger^1^, Jose Eduardo Krieger^1^, Paulo Caleb Junior Lima Santos^2^*

*^1^Laboratory of Genetics and Molecular Cardiology, Heart Institute, University of Sao Paulo Medical School, São Paulo, Brazil.*

*^2^Department of Pharmacology – Escola Paulista de Medicina, Universidade Federal de Sao Paulo EPM-Unifesp, São Paulo, Brazil.*

*Correspondence to: Paulo Caleb J L Santos, PhD

Department of Pharmacology, Escola Paulista de Medicina, Universidade Federal de Sao Paulo EPM-Unifesp Address: 03 de Maio St., 100, Infar, 4th floor, Vila Clementino, São Paulo, SP, Brazil. ZIP Code: 04044-020. E-mail: paulo.caleb@unifesp.br, Phone: +55-11-55764848/Voip 1030.

**Highlights**

The ReHOT study identified 11.7% of RH patients (resistant hypertension).

The genetic ancestry was similar in resistant and nonresistant hypertensive patients.

Increased African ancestry was not associated with RH in this clinical trial.

However, we observed a statistically suggestive association of African ancestry with resistant hypertension in brown patient group.

**Supplementary tables**

**Supplementary table 1.** Allele frequency for ancestry informative markers

| **SNP** | **Chr** | **A1** | **A2** |  | **Frequency of the reference allele**  **(A1)** | | |  | **Population** |
| --- | --- | --- | --- | --- | --- | --- | --- | --- | --- |
|  |  |  |  |  | **European** | **African** | **Amerindian** |  |  |
| rs10493066 | 1 | T | C |  | 0.0336 | 0.6368 | 0.76 |  | EUR |
| rs11804831 | 1 | C | T |  | 0.1621 | 0.91791 | 0.95 |  | EUR |
| rs12119556 | 1 | A | G |  | 0.1166 | 0.0177 | 0.81 |  | NAM |
| rs12135987 | 1 | A | G |  | 0.2806 | 0.1309 | 0.94 |  | NAM |
| rs3827730 | 1 | C | T |  | 0.3406 | 0.06471 | 0.95 |  | NAM |
| rs4844413 | 1 | T | C |  | 0.1344 | 0.1735 | 0.95 |  | NAM |
| rs6429644 | 1 | C | T |  | 0.08135 | 0.8817 | 0.03 |  | AFR |
| rs726570 | 1 | T | C |  | 0.2708 | 0.1162 | 0.94 |  | NAM |
| rs955613 | 1 | T | C |  | 0.2787 | 0.08824 | 0.94 |  | NAM |
| rs10173381 | 2 | G | A |  | 0.1996 | 0.93385 | 0.01 |  | AFR |
| rs10179812 | 2 | G | T |  | 0.08893 | 0.02353 | 0.77 |  | NAM |
| rs10202644 | 2 | T | C |  | 0.1838 | 0.06765 | 0.91 |  | NAM |
| rs10205952 | 2 | G | T |  | 0.2004 | 0.07988 | 0.92 |  | NAM |
| rs10497191 | 2 | T | C |  | 0.1304 | 0.91029 | 0.04 |  | AFR |
| rs11096686 | 2 | C | T |  | 0.1126 | 0.775 | 0.86 |  | EUR |
| rs11123869 | 2 | A | G |  | 0.2451 | 0.04118 | 0.95 |  | NAM |
| rs12470024 | 2 | A | G |  | 0.2233 | 0.03088 | 0.98 |  | NAM |
| rs12471054 | 2 | T | C |  | 0.05138 | 0.05 | 0.81 |  | NAM |
| rs12714093 | 2 | A | G |  | 0.4048 | 0.02515 | 0.98 |  | NAM |
| rs1466020 | 2 | G | A |  | 0.2312 | 0.03392 | 0.94 |  | NAM |
| rs17044697 | 2 | C | T |  | 0.1957 | 0.09265 | 0.93 |  | NAM |
| rs1991600 | 2 | T | C |  | 0.251 | 0.1265 | 0.98 |  | NAM |
| rs2084713 | 2 | A | G |  | 0.3083 | 0.01324 | 0.96 |  | NAM |
| rs2176046 | 2 | A | G |  | 0.05731 | 0.02647 | 0.81 |  | NAM |
| rs2280355 | 2 | A | G |  | 0.1976 | 0.03392 | 0.92 |  | NAM |
| rs260690 | 2 | C | A |  | 0.0498 | 0.6513 | 0.94 |  | EUR |
| rs260714 | 2 | T | C |  | 0.1028 | 0.8038 | 0.94 |  | EUR |
| rs2711302 | 2 | G | A |  | 0.1304 | 0.03557 | 0.83 |  | NAM |
| rs3754675 | 2 | C | T |  | 0.08696 | 0.02663 | 0.92 |  | NAM |
| rs4241135 | 2 | G | A |  | 0.1996 | 0.09412 | 0.92 |  | NAM |
| rs4851839 | 2 | A | G |  | 0.3182 | 0.06618 | 0.99 |  | NAM |
| rs4852013 | 2 | A | G |  | 0.1647 | 0.04853 | 0.86 |  | NAM |
| rs721390 | 2 | T | G |  | 0.249 | 0.08555 | 0.95 |  | NAM |
| rs10935323 | 3 | G | T |  | 0.2668 | 0.07206 | 0.95 |  | NAM |
| rs17008327 | 3 | A | G |  | 0.04348 | 0.05294 | 0.89 |  | NAM |
| rs3870336 | 3 | A | G |  | 0.08696 | 0.1118 | 0.85 |  | NAM |
| rs4855697 | 3 | G | A |  | 0.3775 | 0.08824 | 0.97 |  | NAM |
| rs4955903 | 3 | C | T |  | 0.3182 | 0.06912 | 0.94 |  | NAM |
| rs702032 | 3 | T | C |  | 0.06719 | 0.8426 | 0.67 |  | EUR |
| rs710493 | 3 | G | A |  | 0.01779 | 0.8462 | 0.01 |  | AFR |
| rs7432238 | 3 | G | T |  | 0.07115 | 0.02059 | 0.8 |  | NAM |
| rs868767 | 3 | G | A |  | 0.1056 | 0.02794 | 0.83 |  | NAM |
| rs9871910 | 3 | G | A |  | 0.1957 | 0.125 | 0.91 |  | NAM |
| rs1876495 | 4 | T | C |  | 0.1865 | 0.02206 | 0.88 |  | NAM |
| rs2063393 | 4 | C | T |  | 0.2826 | 0.1003 | 0.93 |  | NAM |
| rs2874414 | 4 | G | A |  | 0.2092 | 0.1756 | 0.94 |  | NAM |
| rs4431170 | 4 | G | A |  | 0.03162 | 0.07647 | 0.81 |  | NAM |
| rs4532240 | 4 | T | G |  | 0.2292 | 0.03687 | 0.9 |  | NAM |
| rs4647693 | 4 | C | T |  | 0.2411 | 0.1879 | 0.95 |  | NAM |
| rs4698702 | 4 | A | C |  | 0.1403 | 0.08133 | 0.94 |  | NAM |
| rs7660290 | 4 | G | T |  | 0.1601 | 0.8912 | 0.88 |  | EUR |
| rs10079352 | 5 | G | A |  | 0.4087 | 0.06471 | 0.98 |  | NAM |
| rs10454965 | 5 | G | A |  | 0.1028 | 0.02212 | 0.88 |  | NAM |
| rs10473594 | 5 | C | T |  | 0.1858 | 0.05 | 0.8878 |  | NAM |
| rs12522914 | 5 | T | C |  | 0.05929 | 0.05882 | 0.84 |  | NAM |
| rs13357733 | 5 | A | G |  | 0.1126 | 0.08382 | 0.88 |  | NAM |
| rs1366220 | 5 | A | G |  | 0.3063 | 0.06471 | 0.93 |  | NAM |
| rs17053011 | 5 | T | G |  | 0.126 | 0.01786 | 0.81 |  | NAM |
| rs1895218 | 5 | A | C |  | 0.2075 | 0.01622 | 0.89 |  | NAM |
| rs1948748 | 5 | C | T |  | 0.1265 | 0.09587 | 0.8878 |  | NAM |
| rs2042314 | 5 | T | C |  | 0.1759 | 0.1559 | 0.93 |  | NAM |
| rs28777 | 5 | C | A |  | 0.0241 | 0.7874 | 0.93 |  | EUR |
| rs4145160 | 5 | A | G |  | 0.08103 | 0.1324 | 0.87 |  | NAM |
| rs4701170 | 5 | A | G |  | 0.166 | 0.08088 | 0.88 |  | NAM |
| rs4868237 | 5 | T | C |  | 0.1126 | 0.01765 | 0.8 |  | NAM |
| rs7728435 | 5 | C | T |  | 0.3992 | 0.06765 | 0.97 |  | NAM |
| rs10484813 | 6 | G | A |  | 0.1512 | 0.05373 | 0.87 |  | NAM |
| rs10945764 | 6 | A | G |  | 0.3413 | 0.04167 | 0.94 |  | NAM |
| rs11153123 | 6 | G | A |  | 0.2945 | 0.1074 | 0.94 |  | NAM |
| rs16888746 | 6 | A | G |  | 0.1344 | 0.04475 | 0.86 |  | NAM |
| rs1744173 | 6 | A | G |  | 0.125 | 0.02206 | 0.91 |  | NAM |
| rs350289 | 6 | A | G |  | 0.1126 | 0.02794 | 0.91 |  | NAM |
| rs3823159 | 6 | G | A |  | 0.004016 | 0.8363 | 0.6 |  | EUR |
| rs4143683 | 6 | C | T |  | 0.2431 | 0.1015 | 0.93 |  | NAM |
| rs4896046 | 6 | T | C |  | 0.2369 | 0.04599 | 0.93 |  | NAM |
| rs6570644 | 6 | G | A |  | 0.256 | 0.93529 | 0.96 |  | EUR |
| rs7753890 | 6 | C | T |  | 0.01581 | 0.8471 | 0.02 |  | AFR |
| rs12701660 | 7 | T | C |  | 0.3281 | 0.2132 | 0.98 |  | NAM |
| rs1550392 | 7 | T | C |  | 0.2004 | 0.06618 | 0.89 |  | NAM |
| rs17166119 | 7 | G | A |  | 0.253 | 0.09292 | 0.91837 |  | NAM |
| rs2040502 | 7 | C | T |  | 0.2925 | 0.1529 | 0.96 |  | NAM |
| rs2471552 | 7 | C | T |  | 0.2095 | 0.08971 | 0.93 |  | NAM |
| rs731257 | 7 | A | G |  | 0.1087 | 0.025 | 0.91 |  | NAM |
| rs978874 | 7 | C | T |  | 0.2866 | 0.1544 | 0.96 |  | NAM |
| rs11776114 | 8 | A | G |  | 0.3142 | 0.04265 | 0.95 |  | NAM |
| rs17072176 | 8 | G | T |  | 0.1502 | 0.01946 | 0.84 |  | NAM |
| rs17807624 | 8 | T | C |  | 0.2945 | 0.1431 | 0.97 |  | NAM |
| rs400404 | 8 | G | T |  | 0.2632 | 0.1627 | 0.98 |  | NAM |
| rs4870958 | 8 | G | A |  | 0.1706 | 0.08676 | 0.89 |  | NAM |
| rs4875427 | 8 | G | A |  | 0.2905 | 0.1195 | 0.94 |  | NAM |
| rs6993205 | 8 | G | A |  | 0.496 | 0.95441 | 1,000 |  | NAM |
| rs7018273 | 8 | G | A |  | 0.01186 | 0.7876 | 1,000 |  | AFR |
| rs896680 | 8 | T | C |  | 0.1687 | 0.1701 | 0.91 |  | NAM |
| rs10812316 | 9 | C | T |  | 0.05534 | 0.7198 | 0.78 |  | EUR |
| rs10962599 | 9 | C | T |  | 0.2411 | 0.95525 | 0.93 |  | EUR |
| rs1412512 | 9 | C | T |  | 0.2016 | 0.2441 | 0.95 |  | NAM |
| rs4135211 | 9 | C | A |  | 0.05929 | 0.005837 | 0.77 |  | NAM |
| rs4877068 | 9 | A | G |  | 0.09881 | 0.0531 | 0.83 |  | NAM |
| rs4979274 | 9 | A | C |  | 0.1206 | 0.02529 | 0.82 |  | NAM |
| rs10821745 | 10 | G | T |  | 0.06126 | 0.08676 | 0.85 |  | NAM |
| rs10994397 | 10 | T | C |  | 0.08103 | 0.08407 | 0.86 |  | NAM |
| rs12220128 | 10 | C | A |  | 0.1265 | 0.08824 | 0.85 |  | NAM |
| rs1419138 | 10 | C | T |  | 0.04941 | 0.8324 | 0.77 |  | EUR |
| rs17130385 | 10 | T | G |  | 0.0751 | 0.0295 | 0.84 |  | NAM |
| rs17143387 | 10 | G | T |  | 0.1621 | 0.05735 | 0.86 |  | NAM |
| rs3998448 | 10 | T | C |  | 0.3267 | 0.02811 | 0.93 |  | NAM |
| rs4746136 | 10 | A | G |  | 0.1508 | 0.01961 | 0.87 |  | NAM |
| rs4880510 | 10 | G | T |  | 0.09486 | 0.08676 | 0.84 |  | NAM |
| rs4880511 | 10 | A | C |  | 0.09486 | 0.02941 | 0.84 |  | NAM |
| rs4918842 | 10 | C | T |  | 0.1364 | 0.08382 | 0.87 |  | NAM |
| rs4948418 | 10 | T | C |  | 0.08135 | 0.01367 | 0.85 |  | NAM |
| rs6482103 | 10 | C | A |  | 0.09091 | 0.08971 | 0.89 |  | NAM |
| rs6585227 | 10 | T | C |  | 0.08893 | 0.2257 | 0.94 |  | NAM |
| rs734241 | 10 | A | G |  | 0.08333 | 0.05 | 0.93 |  | NAM |
| rs7902158 | 10 | G | A |  | 0.2075 | 0.90294 | 0.92 |  | EUR |
| rs7917775 | 10 | C | A |  | 0.06944 | 0.08382 | 0.83 |  | NAM |
| rs7919248 | 10 | G | T |  | 0.08893 | 0.075 | 0.93 |  | NAM |
| rs7923994 | 10 | G | A |  | 0.1719 | 0.3735 | 0.99 |  | NAM |
| rs853577 | 10 | A | G |  | 0.0415 | 0.7035 | 0.77 |  | EUR |
| rs912069 | 10 | C | T |  | 0.2391 | 0.2507 | 0.96 |  | NAM |
| rs1535 | 11 | G | A |  | 0.3142 | 0.1147 | 0.96 |  | NAM |
| rs1561937 | 11 | G | A |  | 0.06746 | 0.8426 | 1,000 |  | AFR |
| rs1638566 | 11 | G | T |  | 0.06522 | 0.8264 | 0.73 |  | EUR |
| rs1638567 | 11 | C | T |  | 0.06522 | 0.8274 | 0.73 |  | EUR |
| rs16921059 | 11 | C | T |  | 0.1957 | 0.1441 | 0.96 |  | NAM |
| rs174546 | 11 | T | C |  | 0.3083 | 0.03382 | 0.96 |  | NAM |
| rs174570 | 11 | T | C |  | 0.123 | 0.01618 | 0.92 |  | NAM |
| rs2241667 | 11 | G | T |  | 0.1957 | 0.07206 | 0.96 |  | NAM |
| rs2403512 | 11 | C | T |  | 0.1779 | 0.03382 | 0.92 |  | NAM |
| rs2619206 | 11 | A | G |  | 0.1235 | 0.06716 | 0.93 |  | NAM |
| rs3736508 | 11 | T | C |  | 0.02191 | 0.007812 | 0.81 |  | NAM |
| rs4309121 | 11 | C | T |  | 0.1581 | 0.01176 | 0.97 |  | NAM |
| rs4963452 | 11 | C | T |  | 0.1295 | 0.1324 | 0.92 |  | NAM |
| rs599190 | 11 | A | G |  | 0.1917 | 0.03687 | 0.94 |  | NAM |
| rs674499 | 11 | A | G |  | 0.07645 | 0.8259 | 0.73 |  | EUR |
| rs10878834 | 12 | C | T |  | 0.08103 | 0.1971 | 0.91 |  | NAM |
| rs11521 | 12 | T | C |  | 0.1957 | 0.08824 | 0.89 |  | NAM |
| rs11612312 | 12 | C | T |  | 0.2036 | 0.04102 | 0.92 |  | NAM |
| rs1397560 | 12 | A | C |  | 0.1567 | 0.1941 | 0.93 |  | NAM |
| rs2051827 | 12 | A | G |  | 0.05138 | 0.04706 | 0.84 |  | NAM |
| rs2052386 | 12 | A | G |  | 0.09486 | 0.1 | 0.91 |  | NAM |
| rs2216437 | 12 | G | A |  | 0.083 | 0.1971 | 0.91 |  | NAM |
| rs3851621 | 12 | C | T |  | 0.06126 | 0.02794 | 0.75 |  | NAM |
| rs1000973 | 13 | A | G |  | 0.2569 | 0.1191 | 0.93 |  | NAM |
| rs17359176 | 13 | A | G |  | 0.082 | 0.01039 | 0.81 |  | NAM |
| rs2166624 | 13 | A | G |  | 0.3794 | 0.03696 | 0.98 |  | NAM |
| rs566996 | 13 | T | C |  | 0.2391 | 0.04412 | 0.89 |  | NAM |
| rs9563982 | 13 | G | A |  | 0.1554 | 0.0374 | 0.84 |  | NAM |
| rs11844034 | 14 | G | A |  | 0.3241 | 0.1696 | 0.98 |  | NAM |
| rs17119138 | 14 | G | A |  | 0.253 | 0.07647 | 0.94 |  | NAM |
| rs17126825 | 14 | A | G |  | 0.1107 | 0.03971 | 0.82 |  | NAM |
| rs1958099 | 14 | G | T |  | 0.1601 | 0.06342 | 0.89 |  | NAM |
| rs7142344 | 14 | T | C |  | 0.09325 | 0.91298 | 0.02 |  | AFR |
| rs810087 | 14 | A | G |  | 0.127 | 0.95 | 0.7653 |  | EUR |
| rs11161328 | 15 | G | A |  | 0.2857 | 0.174 | 0.96 |  | NAM |
| rs11631047 | 15 | T | C |  | 0.2154 | 0.075 | 0.88 |  | NAM |
| rs11637235 | 15 | C | T |  | 0.2273 | 0.96176 | 0.98 |  | EUR |
| rs12594483 | 15 | A | G |  | 0.07708 | 0.94355 | 0.16 |  | AFR |
| rs12594750 | 15 | C | T |  | 0.1166 | 0.08728 | 0.86 |  | NAM |
| rs12917189 | 15 | C | T |  | 0.2174 | 0.94531 | 0.03 |  | AFR |
| rs1448484 | 15 | G | A |  | 0.005917 | 0.8235 | 1,000 |  | AFR |
| rs16951105 | 15 | G | A |  | 0.2451 | 0.05588 | 0.93 |  | NAM |
| rs2122497 | 15 | T | C |  | 0.2242 | 0.06324 | 0.92 |  | NAM |
| rs3884558 | 15 | A | G |  | 0.09486 | 0.226 | 0.92 |  | NAM |
| rs6494489 | 15 | T | C |  | 0.3287 | 0.1519 | 0.97 |  | NAM |
| rs8030587 | 15 | A | G |  | 0.1937 | 0.94853 | 0.03 |  | AFR |
| rs974828 | 15 | T | C |  | 0.083 | 0.91765 | 0.03 |  | AFR |
| rs4141505 | 16 | A | G |  | 0.06126 | 0.09853 | 0.85 |  | NAM |
| rs4889490 | 16 | T | G |  | 0.4071 | 0.03113 | 0.96 |  | NAM |
| rs1049620 | 17 | T | C |  | 0.164 | 0.1553 | 0.91 |  | NAM |
| rs1107704 | 17 | G | A |  | 0.09684 | 0.91176 | 0.07 |  | AFR |
| rs2052074 | 17 | G | A |  | 0.1581 | 0.92206 | 0.03 |  | AFR |
| rs4789659 | 17 | G | A |  | 0.129 | 0.941 | 0.06 |  | AFR |
| rs9916327 | 17 | A | G |  | 0.1621 | 0.04559 | 0.89 |  | NAM |
| rs12104228 | 19 | C | T |  | 0.005929 | 0.7868 | 1,000 |  | AFR |
| rs2074928 | 19 | T | C |  | 0.03755 | 0.005837 | 0.79 |  | NAM |
| rs2313048 | 19 | G | A |  | 0.1719 | 0.03088 | 0.88 |  | NAM |
| rs717225 | 19 | G | A |  | 0.005929 | 0.8162 | 1,000 |  | AFR |
| rs7252511 | 19 | T | C |  | 0.1897 | 0.05294 | 0.9 |  | NAM |
| rs1555133 | 20 | A | G |  | 0.2826 | 0.1309 | 0.94 |  | NAM |
| rs4811693 | 20 | T | C |  | 0.06324 | 0.09265 | 0.82 |  | NAM |
| rs6026036 | 20 | A | G |  | 0.3142 | 0.08088 | 0.94898 |  | NAM |
| rs1626109 | 21 | T | C |  | 0.04382 | 0.8544 | 0.01 |  | AFR |
| rs2248656 | 21 | G | A |  | 0.166 | 0.02794 | 0.84 |  | NAM |
| rs2269160 | 21 | T | G |  | 0.4306 | 0.08676 | 0.98 |  | NAM |
| rs2823850 | 21 | T | C |  | 0.004 | 0.8289 | 0.01 |  | AFR |
| rs2837371 | 21 | A | C |  | 0.1225 | 0.08676 | 0.93 |  | NAM |
| rs2838665 | 21 | A | G |  | 0.2837 | 0.1298 | 0.95 |  | NAM |
| rs717177 | 21 | C | T |  | 0.2668 | 0.1353 | 0.96 |  | NAM |
| rs13054099 | 22 | C | T |  | 0.2826 | 0.02756 | 0.93 |  | NAM |
| rs2024566 | 22 | G | A |  | 0.3532 | 0.1029 | 0.97 |  | NAM |
| rs3927 | 22 | C | T |  | 0.2787 | 0.05 | 0.97 |  | NAM |

**Supplementary table 2.** Association of the genetic ancestry with self-declared race/color

| **Genetic ancestry** |  | **White**  **N = 575** |  | **Brown**  **N = 445** |  | **Black**  **N = 338** |  | **p value** |
| --- | --- | --- | --- | --- | --- | --- | --- | --- |
| European |  | 0.711 |  | 0.510 |  | 0.279 |  | < 2,2e^-16^ |
| African |  | 0.175 |  | 0.350 |  | 0.636 |  | < 2,2e^-17^ |
| Amerindian |  | 0.113 |  | 0.138 |  | 0.084 |  | < 2,2e^-18^ |

**Supplementary table 3.** Association of the genetic ancestry with resistant or nonresistant hypertension among self-reported race/color patient groups

|  |  | | | **White (N= 575)** | | | | |
| --- | --- | --- | --- | --- | --- | --- | --- | --- |
| **Genetic ancestry** | |  | **Nonresistant**  **N = 509** | |  | **Resistant**  **N = 66** |  | **p value** |
| European | |  | 0.711 | |  | 0.709 |  | 0.936 |
| African | |  | 0.174 | |  | 0.174 |  | 0.918 |
| Amerindian | |  | 0.115 | |  | 0.117 |  | 0.710 |
|  |  | | | **Brown (N= 445)** | | | | |
| **Genetic ancestry** | |  | **Nonresistant**  **N = 390** | |  | **Resistant**  **N = 55** |  | **p value** |
| European | |  | 0.516 | |  | 0.471 |  | 0.074 |
| African | |  | 0.346 | |  | 0.393 |  | 0.088 |
| Amerindian | |  | 0.138 | |  | 0.136 |  | 0.880 |
|  |  | | | **Black (N= 338)** | | | | |
| **Genetic ancestry** | |  | **Nonresistant**  **N = 294** | |  | **Resistant**  **N = 44** |  | **p value** |
| European | |  | 0.277 | |  | 0.291 |  | 0.630 |
| African | |  | 0.637 | |  | 0.637 |  | 0.999 |
| Amerindian | |  | 0.086 | |  | 0.072 |  | 0.252 |

**Supplementary table 4.** Association of the African ancestry with resistant or nonresistant hypertension in the brown patient group (N=445)

| **Variables** |  | **Estimate** |  | **Standard error** |  | **p value** |  | **IC (2.5%-97.5%)** |
| --- | --- | --- | --- | --- | --- | --- | --- | --- |
| **Age (years)** |  | 0.016 |  | 0.015 |  | 0.292 |  | (-0.014;0.046) |
| **Gender (male)** |  | -0.599 |  | 0.294 |  | 0.042 |  | (-1.176;-0.022) |
| **Body mass index (kg/m^2^)** |  | 0.005 |  | 0.016 |  | 0.768 |  | (-0.027;0.037) |
| **African Ancestry** |  | 1.424 |  | 0.730 |  | 0.051 |  | (-0.007;2.855) |

**Supplementary table 5.** Association of the general variables with median for the African ancestry in the brown patient group (N=445)

| **Variables** |  | **African ancestry groups** | | |  | **p value** |  | **IC** |
| --- | --- | --- | --- | --- | --- | --- | --- | --- |
|  |  | **≤ median** |  | **> median** |  |  |  |  |
| **Age (years)** |  | 54 |  | 52 |  | 0.743 |  | (-7.000;9.00) |
| **Body mass index (kg/m^2^)** |  | 30.42 |  | 29.92 |  | 0.584 |  | (-4.735;5.372) |
| **Gender (%)** |  |  |  |  |  |  |  |  |
| Men |  | 0.534 |  | 0.466 |  | 0.219 |  | (0.498;1.162) |
| Women |  | 0.474 |  | 0.546 |  | 0.286 |  | (0.853;1.770) |
| **Education level (%)** |  |  |  |  |  |  |  |  |
| Illiterate |  | 0.636 |  | 0.364 |  | 0.131 |  | (0.075;1.310) |
| Elementary school |  | 0.488 |  | 0.512 |  | 0.561 |  | (0.818; 1.478) |
| Higher education |  | 0.535 |  | 0.465 |  | 0.666 |  | (0.293; 1.932) |
| **Alcohol use (%)** |  |  |  |  |  |  |  |  |
| Yes |  | 0.496 |  | 0.504 |  | 0.99 |  | (0.640;1.562) |
| No |  | 0.489 |  | 0.511 |  | 0.98 |  | (0.704;1.420) |
| **Smoker (%)** |  |  |  |  |  |  |  |  |
| Non-smoker |  | 0.530 |  | 0.470 |  | 0.195 |  | (0.552; 1.121) |
| Former smoker |  | 0.474 |  | 0.526 |  | 0.469 |  | (0.742; 2.032) |
| Smoker |  | 0.384 |  | 0.615 |  | 0.069 |  | (0.937;7.250) |
| **Congestive heart failure (%)** |  |  |  |  |  |  |  |  |
| Yes |  | 1 |  | 0 |  | 1 |  | (0.000;166.938) |
| No |  | 0.499 |  | 0.501 |  | 1 |  | (0.768;1.325) |
| **Stroke (%)** |  |  |  |  |  |  |  |  |
| Yes |  | 0.45 |  | 0.55 |  | 0.752 |  | (0.354;6.511) |
| No |  | 0.502 |  | 0.498 |  | 0.945 |  | (0.742;1.297) |
| **Acute myocardial infarction (%)** |  |  |  |  |  |  |  |  |
| Yes |  | 0.429 |  | 0.571 |  | 1 |  | (0.123;30.855) |
| No |  | 0.501 |  | 0.499 |  | 1 |  | (0.753;1.304) |
| **Diabetes mellitus (%)** |  |  |  |  |  |  |  |  |
| Yes |  | 0.492 |  | 0.508 |  | 1 |  | (0.503; 2.253) |
| No |  | 0.501 |  | 0.488 |  | 1 |  | (0.736; 1.329) |
| **Dyslipidemia (%)** |  |  |  |  |  |  |  |  |
| Yes |  | 0.471 |  | 0.529 |  | 0.440 |  | (0.737;2.162) |
| No |  | 0.511 |  | 0.489 |  | 0.637 |  | (0.665;1.264) |
| **Obstructive heart failure (%)** |  |  |  |  |  |  |  |  |
| Yes |  | 1 |  | 0 |  | 1 |  | (0.000; 166.938) |
| No |  | 0.499 |  | 0.501 |  | 1 |  | (0.768; 1.325) |
| **Systolic BP at study entry (mmHg)** |  | 169.25 |  | 171.25 |  | 0.240 |  | (55.62;78.38) |
| **Diastolic BP at study entry (mmHg)** |  | 101 |  | 102.5 |  | 0.714 |  | (-12.250;8.000) |
